# Patients’ experiences with acupuncture for diabetic polyneuropathy as part of a randomized controlled trial (ACUDPN) – a qualitative study

**DOI:** 10.1186/s12906-025-05064-w

**Published:** 2025-09-01

**Authors:** Marie Bolster, Joanna Dietzel, Isabel Valentina Habermann, Sebastian Hörder, Benno Brinkhaus, Barbara Stöckigt

**Affiliations:** 1 Institute of Social Medicine, Epidemiology and Health Economics, Charité- Universitätsmedizin Berlin, Corporate Member of Freie Universität Berlin and Humboldt-Universität zu Berlin, Charitéplatz 1, Berlin, 10117 Germany; 2 Insitute of General Practice and Family Medicine, Charité – Universitätsmedizin Berlin, Corporate Member of Freie Universität Berlin and Humboldt – Universität zu Berlin, Charitéplatz 1, Berlin, 10117 Germany

**Keywords:** Diabetic peripheral neuropathy, Acupuncture, Patient experience, Qualitative study, Symptom management, RCT participation, Mixed-methods

## Abstract

**Background:**

This qualitative study aims to analyse the experiences, perceptions, and motivations of patients with Diabetic Peripheral Neuropathy (DPN), who received acupuncture treatments for the symptoms of DPN as part of a Randomized-Controlled Trial (RCT).

**Methods:**

Semi-structured interviews were carried out with participants of a two-arm RCT as part of a mixed-methods study. The interviews were conducted fact-to-face or via phone using a semi-structured interview guide with questions on living with DPN, the medical care for DPN prior to the study, the acupuncture treatments and the overall trial participation.

**Results:**

In total, 10 participants participated between January – December 2020 in this study. All but one participant had positive experiences with acupuncture and reported a reduction of DPN symptoms. They had varying experiences and needs and adopted a range of different coping mechanisms to manage their symptoms and condition prior to participating in the RCT. Many felt dissatisfied with the lack of current treatment options and insufficient medical care for DPN and wished to reduce medication. Acceptance of acupuncture was high, particularly among those wishing to reduce medication. The findings of the qualitative study are in line with the results of the RCT.

**Conclusions:**

Results the qualitative study indicate that acupuncture could be an acceptable and perceived effective treatment option for DPN patients. Declarations.

**Trial registration:**

RCT: ClinicalTrials.gov NCT03755960. Registered on 11 August 2018.

**Supplementary Information:**

The online version contains supplementary material available at 10.1186/s12906-025-05064-w.

## Introduction

Diabetic Peripheral Neuropathy (DPN) is a neurologic condition affecting around 25% of diabetic patients [1]. Symptoms include numbness, pain and loss of temperature sensation in the legs and feet and are often associated with comorbidities and functional impairments including difficulty of walking, climbing stairs, increased risk of falls, work disability, polypharmacy and higher mortality risk [2, 3]. Treatment most commonly consists of symptomatic pharmacological treatment and rigorous management of the underlying diabetic disease to prevent additional nerve damage [4]. Without adequate management, DPN can result in further complications including diabetic foot syndrome and amputation [3]. As there is currently no curative treatment for neuropathy, DPN can significantly impact the quality of life of patients [5].

Acupuncture, traditionally applied in the context of Chinese Medicine (CM), has been found effective in reducing chronic pain [6] and is recommended in clinical guidelines as a complementary non-pharmacological treatment in chronic pain management [7, 8]. Though more high-quality evidence is needed, clinical trials have also found evidence for the effectiveness of acupuncture in improving symptoms of DPN [9–11]. As a result, acupuncture could potentially be beneficial as a non-pharmacological treatment in the management of DPN.

Though acupuncture for other conditions, like chronic lower back or knee pain, has fallen under coverage of the German statutory insurance since 2007, utilisation has decreased in recent years [12]. In addition to research on the effectiveness of acupuncture, patient perspectives are needed to understand the motivations and experiences of acupuncture utilisation. However, most studies on patient experiences with acupuncture treatment have focused on other chronic conditions [13–15], few studies have assessed the experiences of patients seeking acupuncture treatment for DPN [16].

The present qualitative study was part of a mixed-method research project on the effects of additional acupuncture treatments on symptoms of DPN (ACUDPN). A two-armed, multicentre Randomised-Controlled Trial (RCT) was carried out between February 2019 and April 2021 at Charité – Universitätsmedizin Berlin and at a research and treatment clinic for traditional Chinese medicine in Hamburg, Germany. The intervention group (*n* = 31) received usual care and an additional 12 acupuncture treatments over 8 weeks, while the control group (*n* = 31) received usual care with acupuncture treatments after week 16 from baseline. Quantitative results showed a statistically significant reduction in overall DPN-related complaints and pain in favour of the acupuncture group after 8 weeks. Detailed descriptions of the RCT study design and results have been published elsewhere [17–19].

This qualitative study was nested in the above-mentioned RCT and aimed to assess the subjective experiences, perceptions and motivations of patients who received acupuncture treatment for DPN as part of the RCT. Focus was on the perceptions of and experiences with DPN, with medical care, with the acupuncture treatments and with the participation in the trial. The mixed-methods approach also served to triangulate the results of the validated questionnaires from the RCT and to deepen and complement the findings from the quantitative study.

## Materials and methods

### Study design

We carried out semi-structured interviews with a subgroup of participants from the intervention and control group after completing the acupuncture treatment as part of the RCT.

The self-developed interview guide (Supplement S1) contained questions surrounding four main topics:

- The experiences of living with DPN and its impact on everyday life.
- The perceptions of and satisfaction with medical treatment for DPN prior to participating in the RCT.
- The subjective experience of receiving acupuncture for DPN and the perceived effect on DPN symptoms.
- The experience and perception of the trial participation overall.

Subsequently, the quantitative and qualitative analysis data were triangulated to supplement and deepen the results [20]. The quantitative and qualitative data were integrated data- and outcome-based [21].

### Sampling

**Fig. 1:**
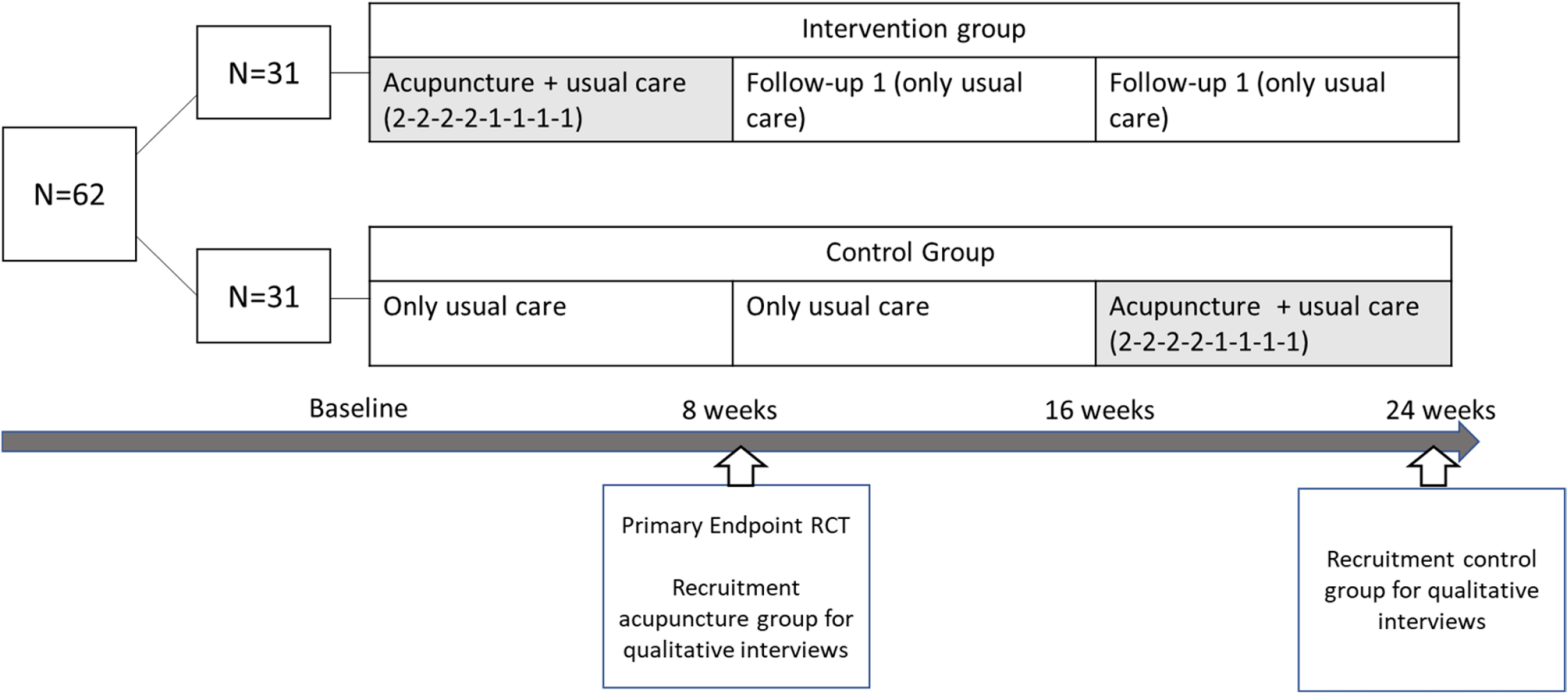
Timeframe of the RCT and recruitment for the qualitative study

All interviewees were participants in the two-arm RCT on the effects of acupuncture on DNP symptoms (Fig. 1). To be eligible to participate in the interview patients had to have participated in either the intervention or control group in the RCT, have completed the last acupuncture treatment at least one week prior, have completed and returned all the questionnaires for the RCT and have provided written consent. Eligible patients were recruited on a rolling basis. To diversify, participants were further selected based on gender, study arm and age. The participants were approached and invited to participate either directly by the treating doctors upon receiving the final acupuncture treatment or contacted by the leading study nurse via phone or email following the final treatment.

### Data collection

Data collection consisted of 10 semi-structured interviews based on an interview guide developed by the research team prior to the first interview. Each participant was interviewed once. Following each interview, the interviewer (M.B.) filled out a study protocol with a summary of the interview content as well as her own thoughts on the interview context and process.

### Data analysis

All interviews were audio-recorded and pseudonymously transcribed verbatim. MaxQDA^®^ Standard 2018 (18.2.4) was used to code and analyse the data.

The data analysis was carried out by the interviewer (M.B.) following the methods of content analysis using deductive and inductive categories [22]. The deductive categories were created based on the structure of the interview guide prior to data analysis. Additional inductive categories were developed during data coding and analysis. The research process was circular and insights from the first interviews and analysis shaped subsequent data collection and analysis.

Triangulation occurred after data collection and analysis was completed for both the RCT and the qualitative study. The overall results of the quantitative study were compared to the overall results of the qualitative study, to identify similarities and discrepancies in findings. Additionally, new and complementary insights from the qualitative study were identified.

### Quality control

The analytic process, categories and methods were continuously discussed among the research team throughout the research process. The interviewer is a female epidemiology/public health researcher (MSc) with prior experience in qualitative research, who had no prior contact with the patients and was not involved with the RCT. Interview participants were informed about the role of the interviewer in the research project prior to the interview. Other members of the research team include three medical doctors with expertise in various Complementary and Integrative Medicine (CIM) methods. One of them also has expertise in qualitative research.

To improve interdisciplinarity and ensure quality, the research process was also presented to and critically discussed with an interdisciplinary qualitative working group (Qualitative Research Network, Charité – Universitätsmedizin Berlin) three times - once during the interview stage (February 2020), once in the data analysis stage (November 2020) and once in the manuscript writing stage (July 2022).

## Results

### Recruitment and sample

Data collection occurred January-December 2020. The first eight interviews were conducted between 13 January- 24th April 2020, after which the RCT had to pause due to containment measures in the context of the SARS-CoV-2 pandemic. The remaining two interviews were conducted in December 2020. Due to pandemic restrictions the time between the participants last acupuncture treatment and the interview ranged from 1 week to 12 months after completing the acupuncture treatment. The first two interviews were led face-to-face at Charité Institute for Social Medicine, Epidemiology and Health Economics; the remaining interviews had to be carried out via phone due to contact restrictions during the SARS-CoV-2 pandemic. Only the interviewer and interviewees were present during the interviews. All patients who were invited to the interview agreed to participate. Interview length varied from ca. 10–30 min. In one interview the recording device only recorded half of the interview due to a technical malfunction. In this case, the transcription was supplemented with the notes by the interviewer right after the interview from the interview protocol. Transcripts were not returned to participants for comment.

Overall, four women and six men between the ages of 60 and 81 were interviewed. Four participants were in the acupuncture group, while six were in the control group (Table 1). While the experiences with DPN and health care were heterogenous between patients, data saturation was achieved in relation to the patients’ experience with routine care and acupuncture for DPN.

**Table 1:** Characteristics of the study participants

No	Sex	Age	Study group	Interval: last acupuncture treatment before interview	Interview mode
1	f	71	Control	1 week	Face to face
2	m	69	Control	3 weeks	Face to face
3	m	60	Acupuncture	17 weeks	Phone
4	m	77	Control	2 weeks	Phone
5	f	81	Acupuncture	24,5 weeks	Phone
6	f	68	Control	6 weeks	Phone
7	m	64	Acupuncture	23,5 weeks	Phone
8	m	64	Control	7,5 weeks	Phone
9	m	81	Acupuncture	12 months	Phone
10	f	72	Control	11 weeks	Phone

### Living with diabetic polyneuropathy

The symptoms of DPN varied between participants. Most mentioned were pain and numbness, mostly in the legs and feet. Other less common symptoms named were loss of temperature sensation, loss of strength/energy, loss of balance, muscle cramps and itching. The symptoms appeared irregularly or with varying intensity for most participants, with some experiencing symptoms predominantly at night. Others reported that symptoms were constant but varied in intensity for some.

When asked about the effect of DPN symptoms on their everyday lives, half of the participants reported not feeling restricted in everyday life or only considered the polyneuropathy a minor nuisance:

“None [restriction] actually, only slightly annoying, but otherwise not at all” [4].

Some participants mentioned limitations in mobility or physical capabilities due to DPN, loss of energy/tiredness, inability to work, negative or depressive thoughts, complete loss of physical and cognitive abilities. A few of the statements by participants were contradictory: while they described restrictions due to the DPN, they did not consider them to impact their everyday life:

“What does that mean for life in general? It doesn’t have such a big impact on my normal life, because I don’t have to walk as much. I usually use my car. But yes, long hikes are no longer possible. But for several years already.” [8].

Half of the interviewees concluded that they had accepted their DPN. Three of them reported not feeling restricted in their everyday lives and seeking distractions from the symptoms, suggesting that DPN did not play a large role in their lives. Nonetheless, several patients took an active involvement in managing their condition, e.g., in researching information on DPN or actively asking their doctors for available treatment options. These contrasting approaches are reflected in some of the patient’s statements:

“Well I accepted it, what can you do?” [1].

“I didn’t get the prospect that I would recover; instead, everyone shook their heads, shrugged their shoulders, and more or less said that it was no longer curable, that it would stay that way and that I would have to deal with the pain. And of course, I didn’t accept that mentally. Not possible, is not possible. Now for me personally.” [7].

The patients had different methods of managing their DPN. Over half of the interviewees mentioned exercise or movement as a way to relieve some of the symptoms, even if only temporary. Many participants also admitted seeking distraction from or not focussing on the symptoms:

“Well, I don’t deal with it all the time. That’s why I can’t say that it somehow restricts me more. I just accept the condition” [9].

Other helpful measures were exchange with peers or support from their social networks or household remedies (e.g., nutritional supplements, cold water). A few participants did not have or know of any ways to help relieve the symptoms, while one person focused on the connection between mind and body.

### Experiences with medical care for DPN prior to the study participation

Medical care and treatment for DPN prior to participating in the study varied between interviewees. Most participants reported receiving pharmacological treatment and podiatry or orthopaedic inlays. Other less common treatments and measures included diagnostic examinations, general diabetes management, electrical/ultrasound therapy, surgery, and rehabilitation.

Participants had mixed experiences with medical care for DPN. Four patients reported feeling well attended by their doctors:

“I got everything I imagined from my family doctor, actually more than I imagined. All the examinations (…) with CT and X-ray and the tube and this and that. They turned me upside down several times (…) Yes, that was already optimal from the conventional medical side.” [7].

Similarly, participants perceived care through podiatrists positively. At the same time, half of the patients did not feel like they received sufficient care and information from their doctors.

“So, the only one who was really concerned, in my opinion, was the podiatrist.” [9].

Almost half the participants reported that while conventional medical care helped lessen their symptoms or at least stopped progression of the condition, it did not suffice to treat their symptoms.

“It helped me a little. Well not really the pain didn’t go away, but I just noticed that when I didn’t take the medication, it got worse.” [8].

Three patients did not consider the conventional medical treatment to improve their condition.

One concern, both from patients who felt well cared for and patients who were dissatisfied with their care, was the lack of treatment options or perspectives for a cure for DPN:

“The diabetologist looks after me very well. [Yet] no one could give me any advice on what to do about polyneuropathy.” [4].

Even though most patients received pharmacological treatment, half of the patients reported that they preferred not to take medication or painkillers and tolerated stronger symptoms in return. Reasons for choosing to reduce medication included fear of losing feeling for the own body, knowing people with addictions to pain medication, or other possible side effects.

### Experiences with acupuncture treatment for DPN

In all but one of the participants, the acupuncture treatment had a perceived positive effect on the DPN symptoms, mainly improvements in sensitivity & mobility and reduction of numbness, pain, itching or burning sensations.

“When I had this acupuncture, I had a day of complete peace. Then I had the feeling that my legs were fine.” [5].

“This improvement came after the first three or four treatments. Then the front foot was free [of numbness], I really felt it and I could also feel that I could move better.” [4].

The interval between acupuncture treatments appeared to affect the treatment effect for some patients, who perceived better effects when treatments were more frequent.

“After the first treatment, the symptoms were already much weaker and I said to one of the doctors treating me that if the treatment took place twice a week, then the result was good and constant, when the intervals became longer, for example only once a week, then the effect was … weaker, or nowadays [17 weeks after the last treatment] I don’t really notice anything anymore.” [3].

The duration of the effect varied, for many of the interviewees it was rather short-term. For two of the patients the effects were still lasting at the time of the interview, however, in those cases the interval between final acupuncture treatment and interview was short (within 1–2 weeks). Two interviewees reported that the effects were persistent, even after several months.

One participant had negative side effects from acupuncture, which triggered pain symptoms from a previously undiagnosed arthritis. Nonetheless, the patient still felt positive about acupuncture after the study. Most participants reported having had positive experiences with acupuncture for other medical conditions prior to participating in the study and the majority of interviewees would consider using acupuncture treatment again.

Generally, patients felt either positive or ambivalent about the sensation of the acupuncture treatment session itself. Any uncomfortable sensations, such as pain from the needling itself, were accepted at the prospect of symptom relief.

### Experience with RCT participation

Almost all the participants took part in the study hoping for symptom improvement. However, most of them were not expecting to be cured or had more modest expectations. Many felt curious to try acupuncture treatment after not having received satisfactory treatment in regular health care or having had a positive experience with acupuncture previously.

“A little bit better, just that I feel a bit better. I didn’t expect them to heal me there, that would have been too much to expect, but that I was better, that was my expectation, and it was fulfilled.” [7].

“Well, because I said to myself, I had assumed that when you take medication, it always has side effects, and I was of the opinion that acupuncture would have no side effects, so I could do it without hesitation. And, as I said, I also had a bit of hope that maybe it would contribute a little bit to the improvement and, as I said, I am also of the opinion that studies are there so that as many participants as possible take part and that was actually also one of the reasons. I didn’t expect any miracles myself.” [9].

The participants were satisfied with the study. All interviewees except one, whose expectations were not met, would participate in the study again and several participants expressed an interest in continuous acupuncture treatment for their DPN.

The most mentioned positive aspects were friendly staff and support, good information on acupuncture and DPN and regularity and punctuality of appointments. All agreed that the study participation and acupuncture treatments were easy to fit into their everyday lives. The participants in the control group did not mind the delayed treatment at the prospect of receiving treatment eventually.

### Triangulation with quantitative data

The overall findings of the qualitative study reflect and complement the quantitative results. Quantitative analysis showed a significant improvement of overall complaints (primary outcome), as well DPN-related pain (VAS Pain, Neuropathy Pain Symptom Inventory (NPSI), affective pain scale (SES), in neurological examination outcomes clinical Total Neuropathy Score (cTNS) and Neuropathy Deficit Symptom Score/(NDS) and Neuropathy Symptom Score (NSS)) and disease specific quality of life (Diabetic Peripheral Neuropathic Pain Impact (DPNPI)) in favour of the acupuncture group at 8 weeks [17, 18]. The positive perception of acupuncture for DPN symptoms in the interviews from both the acupuncture and control group after receiving treatment are in line with these results. Similarly, the reduction in pain was found in both the RCT as well as the qualitative interviews.

Improved sleep was found both in the acupuncture group of the RCT (measured on the DPNPI) and was mentioned by one of the interviewees. Additionally, despite the positive effect of acupuncture on DPN symptoms and disease specific quality of life (DPNPI), there was no effect of acupuncture on overall quality of life for most of the interviewees and no difference between groups in the Global Quality of Life Questionnaire Short Form (SF-12) in the RCT.

There are some discrepancies between qualitative and quantitative results regarding the duration of the treatment effect. Quantitative results persisted with a significant difference until week 16 in the intragroup comparison and in the acupuncture group; the overall complaints were still significantly reduced at week 24, compared to the baseline value [18]. While these results showed long-term effects in the acupuncture group, most of the interviewees mentioned only short-term effects. Only two participants, both from the acupuncture group, mentioned lasting effects in the interviews months after the last treatment. For many interviewees from the control group, however, the interview data cannot be compared to the RCT results, because they were conducted several weeks or months after they completed acupuncture treatment at week 24 and after data collection ended for the RCT.

## Discussion

All but one of the interviewed patients felt an improvement of their DPN symptoms after receiving acupuncture treatment. In terms of the persisting duration of the effect, the results from the qualitative study remain inconclusive. Acupuncture treatment was perceived as a welcome addition to standard care for DPN patients, who often felt that their needs were not entirely met in standard medical care.

### Coping with DPN

DPN and its symptoms, such as pain and numbness, can have a negative impact on the quality of life in patients. Coping strategies as an important way to handle distress and improve the quality of life, can be distinguished into two different approaches: emotion-focused coping, aimed to change the relationship to the stressor, and problem-focused coping, which targets the root of the problem [23].

In our study, participants adopted a range of different physical, behavioural, and cognitive coping strategies to manage their condition and lessen symptoms, which reflects existing research on the experiences of patients with DPN [24–26]. While previous studies have found inconclusive effects of exercise programmes on DPN symptoms and more ambivalent perceptions of DPN patients regarding exercise [24, 25, 27], physical activity and exercise was the most commonly mentioned coping mechanism in our study and well perceived by patients.

In terms of cognitive coping strategies, many of the participants in our study adopted emotion- focused coping strategies, aimed at altering their relationship to their condition and symptoms to reduce impact on their lives [23]. Seeking distraction from the symptoms and acceptance or resignation to the condition were common in our study. While the effectiveness of distraction in altering pain experience in chronic pain patients has not been proven [28], acceptance of pain has been found to increase functioning and improve outcomes for patients [29–31].

Reflecting previous research findings [26], half of the participants in our study did not feel that their DPN symptoms restricted in their everyday lives. Despite the high level of acceptance for the condition and a seemingly low impact on their everyday lives, all patients decided to participate in the acupuncture study hoping to get symptom relief, even when they did not expect to be fully healed. Given the lack of treatment options for DPN, distraction and acceptance or resignation may have been used as a coping mechanism to deal with the loss of control over aspects of their lives. This was reflected in some of the contradictory statements given by the patients in our study, who admitted to avoiding certain activities, e.g. no longer being able to go on walks, but did not feel like they restricted their everyday lives. Yet, when presented with acupuncture as a possible solution, participants adopted a more problem-focused coping mechanism aimed regaining a sense of control and improving the source of their symptoms by participating in the trial [32].

### Experiences with medical care

The results of our study confirm dissatisfaction of patients with routine medical care as a motivation to seek acupuncture treatment [13]. International studies have found that DPN is frequently inadequately managed and undertreated in medical care due to a lack of awareness by physicians and patients of the condition, under- or low dosage prescription of medication and low treatment adherence [33, 34]. Correspondingly, many of our study participants, reported frustration with the current treatment options and insufficient care and symptom relief for DPN.

Though pharmacotherapy was the most common treatment for DPN addressed, many patients preferred not taking medication and tolerated stronger symptoms return. This reluctance in older patients or those with chronic conditions to take medication, e.g. due to fear of side effects or dependencies or lack of effectiveness, has been reported before [14, 35]. Low acceptability of pharmacological treatment by patients might be a contributor to a low treatment adherence found in DPN patients [33].

Reducing medication has also been shown to be a motivation for patients with various chronic diseases and pain to seek acupuncture treatment [13–15]. Diabetic patients are often subject to polypharmacy. The low acceptance of pharmacological treatment in our study could in part be caused by self-selection of the patients participating in the acupuncture trial, many of whom have had previous experience with acupuncture, and thus might not be representative for all DPN patients. However, for those patients wishing to reduce medication or those who preferred alternatives to pharmacological treatments, acupuncture seemed to provide an acceptable alternative or complementary treatment for DPN symptoms.

### Experience of acupuncture and effect on DPN

Though the effectiveness of acupuncture in DPN is not yet conclusive, our study results contribute to a growing body research, including the quantitative results from our RCT, which found positive effects of acupuncture on symptom improvements for DPN [9, 18, 36, 37]. Most of the patients reported symptom improvement after receiving the acupuncture treatment, an effect that was perceived mostly short-term and improved with regular treatments. While there is no established dose to achieve optimal effects in most clinical trials on acupuncture for chronic pain, there are indications that more frequent acupuncture treatments improve patient outcomes and are an important aspect of patient satisfaction [13, 38–40].

An important factor for the patients were the contextual factors of the treatment setting. Contextual factors, such as communication and the relationship between patient and medical practitioner, have been identified as influencing health outcomes both in regular care and in CIM [13, 15, 41, 42]. When asked about the experience of receiving acupuncture treatments, most of the interviewees commented on the experience of participating in the study (atmosphere, friendliness of staff, effect of acupuncture treatment) rather than the treatment process itself. Contextual factors like attention, atmosphere and treatment effect appeared to be more memorable to patients than the actual sensations of receiving acupuncture, which they only discussed when prompted. The importance for patients to being tended to and cared for is also reflected in the positive perceptions of podiatrists.

The satisfaction with the RCT participation stands in contrast to the dissatisfaction many patients had with medical care for DPN prior to participating in the study, where many felt they did not receive effective treatment options, information or satisfactory care. Coming from a place of desperation and seeking a sense of hope in face of chronic disease, has previously been identified as a motivation for chronic patients to try acupuncture [15, 16]. Similarly, most patients turned to acupuncture out of a lack of treatment options in regular medical care, curiosity, a sense of nothing to lose or a positive experience with previous acupuncture. Though they did not necessarily expect to be cured, they did hope to get some symptom relief.

### Triangulation with quantitative data

The qualitative results complement the quantitative results in several aspects: The positive effect of acupuncture on DPN symptoms from the control group in the interviews and the questionnaires and neurological examinations confirm the favourable results in the acupuncture group in the RCT. They give an additional insight into the effects of acupuncture treatment of the control group after the data collection for the RCT ended. The qualitative results provided a larger and more in depth understanding of the experiences of patients living with DPN. Findings from the qualitative study identified the different coping strategies adopted by patients, unmet medical care needs as well as motivations to seek acupuncture treatment, which were topics not covered by the questionnaires. Lastly, the results shed light not only on the outcomes but also on the experience of the acupuncture treatments, which was not addressed in the RCT. They highlight the importance of contextual factors such as friendliness of staff, attentiveness, punctuality and setting/atmosphere as important features in the perception of acupuncture treatment for the patients. As time and level of attention given to the two treatment groups differed during the RCT, with the control group only receiving usual care up to week 16, those contextual factors cannot be excluded as an influencing factor on treatment effect. An important finding was that acupuncture was an acceptable treatment, particularly for those patients, who refused or wished to reduce medication.

### Implications for practice

Due to the chronic nature of the condition, our study shows that the needs of DPN patients are complex and not currently addressed sufficiently in usual DPN care. To better address the needs of patients, multidisciplinary DPN management that complements traditional pharmacological treatment and diabetes management could benefit DPN patients. Our mixed-method study contributes to a growing body of research that finds positive effects of acupuncture on DPN symptoms and identifies it as a well-accepted and feasible treatment for DPN patients, particularly those who wish to reduce medication. As such it provides a complementary treatment that could be integrated in DPN care.

### Strengths and limitations of the study

The mixed-method design and triangulation of quantitative and qualitative results in this study allowed for both confirmation of quantitative results as well as identification of new topics that were not covered in the quantitative study. It also allowed shedding more light on the subjective and personal experiences with acupuncture of the control group.

Unfortunately, especially due to the SARS-CoV-2 pandemic, a few adjustments had to be made throughout the study process. First, due to contact restrictions all but two interviews had to be conducted via phone rather than face-to-face. This could have affected the relation between interviewer and patient’s and did not allow for the interpretation of physical cues of body language [43]. At the same time, phone interviews from their homes may have provided a more private and comfortable setting for the participants, which might have helped patients to speak and voice opinions more openly. Secondly, due to a pause of the RCT, some of the participants were interviewed up to 12 months after the acupuncture treatment had ended and the sample size was limited to only 10 participants. This could have increased the risk of a recall bias in patients who had received their last acupuncture treatment up to one year before the interview [44], yet as a strength, it also brought in more variation in the study sample.

Overall, the results of this study reflect the subjective experience of patients with DPN who participated in an RCT to investigate the effect of acupuncture on DPN symptoms. Due to this self-selection, this sample shares some characteristics. For example, the participation in the RCT assumes an openness and interest in acupuncture or CIM and many of the patients have had previous experience with acupuncture. As a result, the subjective experiences and perceptions of DPN patients within this study should be interpreted in this context. Additionally, participation in the qualitative study was voluntary, so there is a risk of self-selection bias, whereby patients who have a positive acupuncture experience in the RCT are more likely to participate in the qualitative study. However, considering that recruitment was rolling, none of the participants refused the interview and a patient with a negative experience participated, this risk should be minimal. Additionally, the overall positive experience of the qualitative study reflects the experience of the larger sample of the RCT.

## Conclusions

The overall perception of acupuncture treatments for DPN was positive. Almost all patients reported an improvement of symptoms, mainly pain and numbness, although the duration of the effect varied or was inconclusive. As many patients were dissatisfied with standard care for DPN and a lack of treatment options or perspective, acupuncture was a welcome and acceptable complementary additional treatment, particularly for those refusing or wishing to reduce medication. The qualitative results compliment the quantitative results from the RCT, which found symptom improvements in DPN symptoms lasting until 24 weeks from baseline. In addition, the qualitative study found new insights on the subjective experiences and perceptions of DPN patients, regarding their coping mechanisms, unmet medical needs and motivations to seek additional acupuncture treatment. Results the qualitative study indicate that acupuncture could be an acceptable and perceived effective treatment option for DPN patients.

## Supplementary Information

Supplementary Material 1.

## Data Availability

No datasets were generated or analysed during the current study. To ensure data protection the interview transcripts will not be made publicly available. The interview guide is publicly available in the supplementary material.

## Abbreviations

CIM: Complementary and Integrative Medicine
CM: Chinese Medicine
cTNS: Clinical Total Neuropathy Score
DPN: Diabetic Peripheral Neuropathy
DPNPI: Diabetic Peripheral Neuropathic Pain Impact
NDS: Neuropathy Deficit Symptom Score
NPSI: Neuropathy Pain Symptom Inventory
NSS: Neuropathy Symptom Score
RCT: Randomised-Controlled Trial
SES: Schmerzempfindungsskala (Affective Pain Scale)
VAS: Visual Analog Scale
