# Supplementary material for: Patients’ experiences with acupuncture for diabetic polyneuropathy as part of a randomized controlled trial (ACUDPN) – a qualitative study
Source: BMC Complement Med Ther. 2025 Sep 1;25:317. doi: 10.1186/s12906-025-05064-w (PMC12400560; doi:10.1186/s12906-025-05064-w)
Supplement: Supplementary file 1 — Supplementary Material 1. [file 12906_2025_5064_MOESM1_ESM.doc]

**Supplementary Material**

**Manuscript Titel: „Patients’ experiences with acupuncture for diabetic polyneuropathy as part of a randomized controlled trial (ACUDPN) – A qualitative study”**

**Autors: Marie Bolster^1,2^; Joanna Dietzel^1^; Isabel Habermann^1^; Sebastian Hörder^1^; Benno Brinkhaus^1^; Barbara Stöckigt^1^**

**^1^** Institute of Social Medicine, Epidemiology and Health Economics, Charité-Universitätsmedizin Berlin, Corporate Member of Freie Universität Berlin, Humboldt-Universität zu Berlin, and Berlin Institute of Health, Luisenstr. 57, 10117 Berlin, Germany

**^2^**Insitute of General Practice and Family Medicine, Charité – Universitätsmedizin Berlin, Corporate Member of Freie Universität Berlin, Humboldt – Universität zu Berlin, and Berlin Institute of Health, Charitéplatz 1, 10117 Berlin, Germany

Corresponding author: Marie Bolster (marie.bolster@charite.de )

Funding: The trial was an investigator-initiated trial and was partially funded by a research grant for J.D. from the Veronica and Karl Carstens Foundation, Am Deimelsberg, Essen, Germany (grant no. KVC 0/099/2018).

**S1: Interview guide ACUDPN**

The numbered questions/prompts are key questions; the hyphenated/cursive questions may (but not mandatorily) be used for further questions if the topic is not addressed in the answer or if the interview is faltering.

**Introduction**

I am very happy that you agreed to be interviewed for this study. Do you have any questions before we begin?

**Start recording device**

**Experience with polyneuropathy**

1. First, I would like to learn more about your existing polyneuropathy (DPN). Please describe to me what kind of impact your condition has on your life.

- *What does your everyday life with polyneuropathy look like?*
- *Can you elaborate a little using examples?*

1. How did you cope/are you coping with your polyneuropathy so far?

- *What helps you/doesn’t help you?*
- *You mentioned_____ - can you tell me more about it? Did that help you? How so?*

**Medical care**

1. How did you perceive the medical care for your DPN prior to participating in the study?

- *How was your DPN treated?*
- *Did it help you? How so?*
- *Can you describe this in a bit more detail?*

**Acupuncture treatment**

1. You have received acupuncture treatment for your polyneuropathy over several weeks. Please tell me how you experienced this treatment.

**-** *can you describe this in more detail using examples?*

*- was there anything that was particularly pleasant or unpleasant for you?*

*- How did you feel immediately after the acupuncture sessions?*

*- How long did this feeling last?*

1. Did the acupuncture have any effects on your DPN?

- *If so, which ones?*
- *Can you describe this in more detail using examples?*
- *How long did these effects last?*
- *Has this affected your everyday life? If so, how?*
- *Possible question: Your treatment had to be paused due to the covid pandemic. How was that for you? Did the pause in treatment affect your symptoms?*

1. Did you feel the acupuncture affected other areas in your life?

- *Can you describe this in more detail/elaborate?*
- *You mentioned______ earlier – can you describe this in more detail/elaborate?*

1. Could you imagine to get acupuncture treatments for other physical ailments?

- *Why? Why not?*

**Study participation**

1. What was your motivation to participate in the study?

- *What were your expectations in participating in the study?*

1. How was it for you to participate in the study?

- *Where there any particularly positive or negative aspects?*
- *For control group: How was it for you to be assigned to the control group?*
- *Would you change anything about the study? If so, what would you change?*

1. How did you find the questionnaires?

- *Did you feel the questionnaires covered relevant/important aspects of your life, your symptoms and the treatment?*

1. In hindsight, would you participate in the study again?

- *Why? Why not?*

**Conclusion**

1. Would you like to add anything that is important to you that we have not addressed today?
2. Do you have any question or comments that you would like to share?

Thank you very much for taking the time to participate in the interview.
